# Metagenomic Insights into the Sewage RNA Virosphere of a Large City

**DOI:** 10.3390/v12091050

**Published:** 2020-09-21

**Authors:** Sergio Guajardo-Leiva, Jonás Chnaiderman, Aldo Gaggero, Beatriz Díez

**Affiliations:** 1 Department of Molecular Genetics and Microbiology, Pontificia Universidad Católica de Chile, Santiago 8331150, Chile; guajardo.sergio@gmail.com; 2 Programa de Virología, Instituto de Ciencias Biomédicas, Facultad de Medicina, Universidad de Chile, Santiago 8380453, Chile; jchnaiderman@med.uchile.cl; 3 Center for Climate and Resilience Research (CR)2, Santiago 8370449, Chile

**Keywords:** sewage RNA virosphere, viral metagenomics, wastewater, *Picobirnavirus*, *Rotavirus*

## Abstract

Sewage-associated viruses can cause several human and animal diseases, such as gastroenteritis, hepatitis, and respiratory infections. Therefore, their detection in wastewater can reflect current infections within the source population. To date, no viral study has been performed using the sewage of any large South American city. In this study, we used viral metagenomics to obtain a single sample snapshot of the RNA virosphere in the wastewater from Santiago de Chile, the seventh largest city in the Americas. Despite the overrepresentation of dsRNA viruses, our results show that Santiago’s sewage RNA virosphere was composed mostly of unknown sequences (88%), while known viral sequences were dominated by viruses that infect bacteria (60%), invertebrates (37%) and humans (2.4%). Interestingly, we discovered three novel genogroups within the *Picobirnaviridae* family that can fill major gaps in this taxa’s evolutionary history. We also demonstrated the dominance of emerging *Rotavirus* genotypes, such as G8 and G6, that have displaced other classical genotypes, which is consistent with recent clinical reports. This study supports the usefulness of sewage viral metagenomics for public health surveillance. Moreover, it demonstrates the need to monitor the viral component during the wastewater treatment and recycling process, where this virome can constitute a reservoir of human pathogens.

## 1. Introduction

Viruses are the most abundant biological entities on Earth, with an estimated 10^31^ particles worldwide [1]. Urban environments impacted by human activity, such as wastewater treatment plants (WWTPs) and sewage are not an exception. Sewage and WWTPs form an ecosystem that supports thriving microbial communities (prokaryotic and eukaryotic), plants and animals, such as rodents, birds and bats [2]. In these environments, viruses associated with the biological waste of a city are mixed with viruses from all the organisms living in the WWTP, thus forming an untapped source of viral diversity [2,3]. In general, the sewage virosphere can be considered a mixture of human viruses excreted in the feces, urine and skin peeling, and viruses from animals, invertebrates, plants, fungi and bacteria [4,5].

Sewage has been historically used to monitor known human viral pathogens, such as noroviruses, hepatitis viruses, enteroviruses, rotaviruses and adenoviruses [6,7]. The presence of viral pathogens in wastewater reflects ongoing infections being transmitted in the human population served by the given WWTP [2,3,4]. Likewise, sewage can reveal new and unknown viral genomes that could, in the future, be associated with idiopathic human diseases [3].

Nowadays, decreased water availability due to global warming and increased human water consumption have turned water scarcity into a cyclical problem. To solve this, recycled water derived from WWTP effluent has been intensively used for industrial operations, agricultural irrigation and even recreational activities [8]. In this way, recycling of treated sewage generates a potential public health risk, as well as a critical risk for agricultural and animal production industries, due to insufficient removal of pathogenic viruses [6,8,9]. Current regulations have promoted improved treatment guidelines, which now combine mechanical, biological and chemical processes, such as flocculation, sedimentation, filtration, chlorination and UV-radiation [6,8,9]. These treatments have significantly reduced microbiological contamination by inactivating and removing bacteria and protozoa, but they have little effect on human viruses, such as adenoviruses and enteroviruses, which are later dispersed in effluent waters [8,9,10].

In Latin America, the sanitary systems, which are still under development, have a low sewage treatment coverage (30%), generating a potential health risk for human and animal populations [11]. Chile is a middle-income country with a population of 19 million inhabitants, of which approximately 7 million inhabitants are concentrated in the capital, Santiago de Chile, making it the seventh largest city in the Americas. Most of Santiago’s sewage is decontaminated by three WWTPs: El Trebal, La Florida and La Farfana. El Trebal serves 3.2 million equivalent inhabitants, and water decontaminated by this WWTP provides irrigation for 57,800 agricultural hectares of land that is mainly used for the production of fruits and vegetables consumed in Santiago and exported according to international environmental standards.

The sewage virosphere has been monitored world-wide using molecular techniques, such as PCR and quantitative PCR (qPCR) [5,7]. These methods can only provide information about the presence and abundance of known and characterized viruses because there is no universal marker for viruses, such as the 16S rRNA gene for bacteria [4,5]. Despite the increasing application of high-throughput sequencing techniques (HTS) for viral metagenomics, their use in identifying viruses in sewage has not been well explored [4,5,11]. However, the few existing studies have demonstrated that the study of the viral metagenomics of sewage is an excellent tool to monitor, identify and explore the diversity of the viral communities circulating among human and livestock populations [2,4,5,7,11,12]. This is especially important for RNA viruses that, in the last decade and along with viral metagenomics, have undergone a revolution in terms of their discovery, thereby contributing to our understanding of viral diversity [13]. 

To our knowledge, few studies [4,7] have used viral metagenomics to investigate RNA viral communities in sewage around the world, none of which have been conducted in Latin America. With this in mind, we conducted a pilot study to investigate the RNA virosphere using a single sample equivalent to the sewage treated during one day in Santiago (El Trebal WWTP) using viral metagenomics.

Our main results, despite bias related to the overrepresentation of dsRNA viruses (in comparison to other studies), show that the RNA virosphere of El Trebal is mainly composed of unknown sequences (microbial and viral dark-matter). The known viral sequence fraction was dominated by viruses that infect bacteria and invertebrate hosts. A high diversity and novelty were discovered within the *Picobirnaviridae* family, which can fill major gaps in the evolutionary history of this group. Likewise, we unveiled abundant and emergent *Rotavirus A* genotypes never before recorded in Chile, thus representing changes in the prevalence of the classical genotypes. 

The latter discovery provides evidence for the benefits of using viral metagenomics to aid public health surveillance based on excreted viruses in sewage. Additionally, this study reveals the importance of analyzing viral dark matter through self-clustering of the sequences independent of their direct comparison to databases, which can result in the discovery and classification of new viral sequences.

## 2. Materials and Methods 

### 2.1. Study Site and Sample Preparation

**Figure 1:**
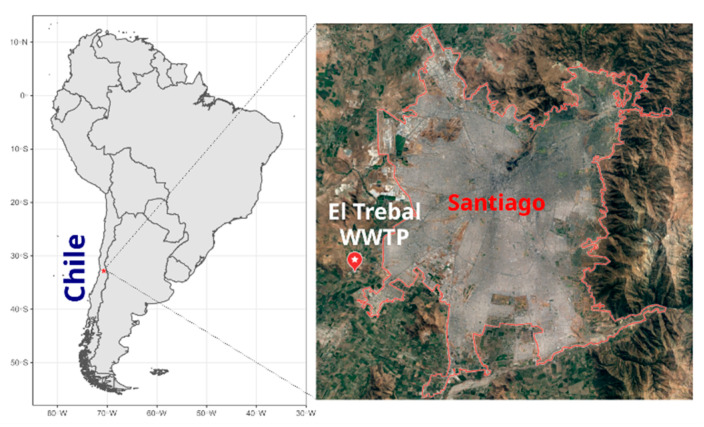
Map of el Trebal Wastewater Treatment Plant location in Santiago de Chile. El Trebal wastewater treatment plant (WWTP) is indicated by a white-star red baloon.

El Trebal (hereafter referred to as Trebal) is a WWTP (33°32′28.5″ S 70°50′08.2″ W) located in Santiago, Chile (Figure 1), that serves a population equivalent to 3.2 million inhabitants. A composite sample (1 L), representing 24 h of raw sewage, was obtained on 5 Jun 2017. The sample was sequentially filtered through 8- and 3-μm pore size polycarbonate filters (Isopore, 47 mm diameter, Millipore, Milford, MA, USA) using a Swinex filter holder (Millipore) and then a 0.22-μm pore size filter (Sterivex PES, Millipore). Particles in the 0.22-µm filtrate were concentrated by ultracentrifugation to a final volume of approximately 1 mL, as described in [14]. Briefly, the 0.22-µm filtered sample was centrifuged at 100,000× *g* for 1 h. The pellet containing viral particles was resuspended in glycine buffer and then incubated on ice for 30 min. Finally, after an additional ultracentrifugation at 100,000× *g* for 1 h, viruses were recovered by resuspending the viral pellet in 1 mL of PBS.

### 2.2. RNA Extraction and High-Throughput Sequencing

The resuspended viral particles (1 mL) were treated with DNase I (600 U) to remove the remaining free DNA from the cellular fraction. The mixture was incubated for 1 h at 37 °C, followed by inactivation at 75 °C for 10 min. Viral RNA was extracted using the High Pure Viral RNA kit (Roche, Basel, Switzerland) according to the manufacturer’s instructions, but without the use of the poly(A) carrier. 

Bacterial DNA contamination was checked by 16S rRNA gene PCR amplification using a universal bacterial primer set (515F: 5′-GTGYCAGCMGCCGCGGTAA-3′ and 806R: 5′-GGACTACNVGGGTWTCTAAT-3′) https://earthmicrobiome.org/protocols-and-standards/16s/. Bacterial (*E. coli* JM109) DNA was used as a PCR spike control to check for PCR inhibition of viral RNA. The purified RNA sample was then sequenced using Illumina HiSeq technology (Roy J. Carver Biotechnology Center, Urbana, IL, USA). Briefly, the RNAseq library was prepared with the Illumina TruSeq Stranded mRNA Sample Prep kit (Illumina, San Diego, CA, USA). The library was quantitated by qPCR and sequenced from one end of the fragment in a single lane for 151 cycles on a HiSeq 4000. Fastq files were generated and demultiplexed with the bcl2fastq v2.17.1.14 Conversion Software (Illumina).

### 2.3. Viral RNA Metagenome Processing

Raw metagenomic reads were quality filtered using Cutadapt v2.1 [15], leaving only sequences longer than 50 bp (-m 50), and conducting 3′ end trimming for bases with a quality below 30 (-q 30) and hard clipping of the first five leftmost bases (-u 5). Finally, sequences representing simple repetitions, which are usually due to sequencing errors, were removed using PRINSEQ v0.20.4 [16] at a DUST threshold of 7 (-lc_method dust, -lc_threshold 7). Details of the obtained sequences are shown in Supplementary Table S1.

Viral RNA metagenomes were assembled using De Bruijn graphs, as implemented in the MEGAHIT v1.2.9 [17] and IDBA-UD v1.13 assemblers [18] in metagenomic mode. Only contig sequences >200 pb were further analyzed. Both assemblies were merged by clustering contigs at 100% identity and 100% coverage of the shortest sequence, leaving the largest contig using the NUCmer algorithm implemented in MUMmer v3 [19]. After assembly, Prodigal software [20] was used to predict protein-coding regions, with options (-p meta -n).

### 2.4. Taxonomic Assignment of Viral Proteins

The predicted proteins were aligned against the NCBI nr database using DIAMOND v2.0.4 [21] (--*e*-value 0.00001) and parsed using the lowest common ancestor algorithm in MEGAN 6 [22] (LCA score = 50) using NCBI taxonomy tree to obtain the taxonomic annotation of each viral protein. Species classification of viral proteins was used to infer putative hosts based on the Virus–Host DB [23], as described in [24].

The abundance of mapped proteins was quantified through read recruitment via Bowtie2 [25], with parameters (-end-to-end-very sensitive-N 1). The resulting SAM file was parsed by the BBmap pileup script (Bushnell B.—sourceforge.net/projects/bbmap/) and the relative protein abundances were normalized by gene length.

### 2.5. RNA-Dependent RNA Polymerase Analyses

The predicted proteins were functionally annotated using the Pfam v32 database [26] through hmmscan options (--cut_ga) implemented on HMMER3 [27]. Proteins annotated as RNA-dependent RNA polymerase (RdRP; 2266 predicted proteins) were taxonomically identified as described before, and the species classification was used to infer putative hosts. Pairwise genetic distances were calculated for Trebal RdRP nucleotide sequences and RefSeq (release 97) using word-based alignment-free distances implemented on Alfree software [28], with options (-s 2-d braycurtis-v counts).

The pairwise distances between samples were analyzed by hierarchical clustering (hclust function in R) using a minimal increase in the sum of squares method (Ward’s method). The resulting dendrogram was cut (cutree function in R) into 12 groups that represent the main clusters based on the “unrooted” dendrogram representation.

### 2.6. Phylogenetic Analysis of Picobirnaviruses and Ribosomal Binding Site Prediction

The Trebal proteins annotated as RdRP (>450 aa) using the Pfam database [26] and classified as *Picobirnaviridae* were aligned by MAFFT v7 [29] using default parameters, with the option --globalpair. A phylogenetic RdRP gene tree was constructed using the maximum likelihood method implemented with IQtree (-bb 10,000-nm 10,000-alrt 1000-abayes) and 10,000 ultrafast bootstraps to evaluate branch robustness [30]. The amino acid substitution model used in the phylogenetic analysis (VT + F + I + G4) was determined by modelFinder [31]. Reference sequences were obtained from NCBI RefSeq based on the phylogenetic analysis of the *Picobirnaviridae* family from the International Committee on Taxonomy of Viruses (ICTV) [32], and a reference genome of the genus *Alphapartitivirus* was used as an outgroup.

A ribosomal binding site (RBS) prediction of the 31 RdRP-predicted proteins used in the phylogenetic analysis was performed using Prodigal software [20], as described before.

### 2.7. Human Rotavirus Classification

Nucleotide sequences of Trebal proteins annotated as *Rotavirus* VP4, VP6 and VP7 using the Pfam database [26] were aligned against the NCBI nt database using BLASTn [33]. The best hit (*e*-value < 0.00000001) classification of each nucleotide sequence was used to assign *Rotavirus* species and type (G and P).

### 2.8. Data Availability

Raw sequences are available under NCBI SRA BioProject PRJNA648644.

Assembled contigs and their annotation are available in the MG-RAST server under the accession number mgm4904696.3 and can be accessed at the following link https://www.mg-rast.org/linkin.cgi?project=mgp95699.

## 3. Results and Discussion

### 3.1. Predicted Protein-Based Analysis of Trebal RNA Viral Metagenome 

The Trebal RNA viral fraction, sequenced on the Illumina HiSeq platform, yielded ~45 M high-quality reads (Supplementary Table S1). Assembly of the RNA metagenomic reads resulted in 62,164 contigs, which included 34.8 M reads and 72,313 predicted proteins. As with most viral metagenomes, only a small fraction (~12%) of the viral proteins matched to large databases [34,35] such as NCBI nr, representing ~15% of the assembled reads. The high number of unmapped contigs (85% of the assembled reads) are most likely derived from novel and uncharacterized microbial and viral sequences, as has been observed in other wastewater studies [2,7].

**Figure 2:**
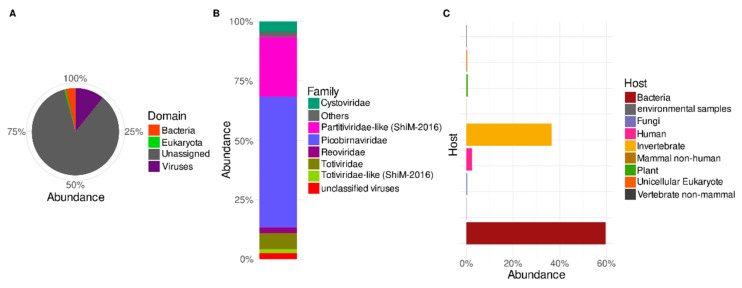
Relative abundances of predicted protein sequences in Trebal viral RNA metagenome, classified by LCA algorithm trough local alignment to NCBI nr database. (**A**) Domain level, and (**B**) Family level for sequences classified as Virus in A. (**C**) Putative host for sequences classified as Virus in A. Sequences were normalized by protein length.

A taxonomic survey of the predicted proteins shows that the known Trebal reads (Figure 2A) were assigned mostly to the Virus domain (72%), followed by Bacteria (24%) and Eukarya (2%). Cellular contamination of viral enriched fractions is a common feature of viral metagenomes [36,37,38]. In this study, this could correspond to cellular transcripts, probably due to the lack of RNAse treatment during nucleic acid preparation, or the presence of some residual DNA after the DNase treatment, although the 16S rRNA gene was not amplified by PCR. Viral sequences can also be misannotated to homologous cellular genes [36,39], which relies on the low number and diversity of viral sequences in the databases. Additionally, horizontal gene transfer between viral and host genomes can lead to incorrect annotation based on the closest homologous sequences [36,39]. Misleading annotation is a frequent phenomenon in underexplored communities, such as environmental RNA viruses where new schemes of classification are needed [13].

In further sections, we only analyze proteins classified as having viral origin. Most of the viral proteins in the Trebal RNA viral metagenome (Figure 2B) were classified as belonging to the *Picobirnaviridae* family (55%), followed by *Partitiviridae* (~25%) and other families, such as *Totiviridae* (~7%), *Cystoviridae* (~4%) and *Reoviridae* (~2%). In summary, most of classified viral sequences (97%) belong to families comprising Group III (dsRNA viruses) of the Baltimore classification. Nevertheless, ssRNA (e.g., *Virgaviridae* and *Leviviridae)*, dsDNA (e.g., *Myoviridae* and *Siphoviridae*), and ssDNA (*Microviridae*) viral families were detected, but their relative abundances were below 1% (Supplementary Table S2). It is important to emphasize that since we did not perform any amplification step (e.g., MDA) before library construction, it is not expected that relative viral abundances were affected by amplification bias in any steps before library preparation. Thus, a possible explanation for the low abundance of ssRNA viruses is that the inclusion of an inactivation step using DNase at 75 °C could potentially enhance the effect of natural RNases present in the sample, as has been described before [7]. However, this must be confirmed experimentally using an ssRNA virus as spike control subjected to the indicated conditions in the same type of matrix. Despite possibly missing some viral types during the extraction procedure, the characterized viral RNA metagenome still harbors a vast diversity of viruses within each family.

In general, RNA viral families identified here have been identified in other previous studies that describe the RNA virus diversity of wastewater [2,5,7,40,41,42]. However, the viral community’s specific taxonomic composition in the Trebal has not been reported in other sewage studies. Picobirnaviruses (PBVs) were dominant in sewage influent samples from Wales, UK [7]. Likewise, PBVs were prevalent in sewage samples across the USA [40] and have been proposed as a potential marker of fecal pollution [41]. Viral sequences identified as *Partitiviridae*-like viruses included in the “unclassified RNA viruses ShiM-2016” category in the NCBI taxonomy (~25% abundance; Figure 2B) and *Totiviriade* family were also highly abundant in treated and untreated sewage samples from the EU [5,7]. The *Cystoviridae* family (~4% abundance; Figure 2B) is the only ICTV-recognized bacteriophage family detected at more than 1% abundance. These bacteriophages are known to be abundant in the gastrointestinal tract of vertebrates and as part of raw sewage samples [42]. Only recently was this family reported in metagenomic assessments of sewage samples using previously published viral metagenomes from Pittsburgh, Barcelona and Addis Ababa [2,42]. Finally, the *Reoviridae* family, represented by the genus *Rotavirus*, accounted for ~2.4% abundance. These human pathogenic viruses are routinely found in raw sewage samples using amplification techniques such as RT-PCR and RT-qPCR [14,40]; however, they have been detected by HTS in a recent investigation in Wales, UK [7]. Since some human pathogenic viruses are seasonal (e.g., *Rotavirus* and *Norovirus*), their presence in wastewater can also vary, which could explain the absence of *Rotavirus* in previous metagenomic surveys of sewage [8]. 

### 3.2. Putative Hosts of Sewage RNA Viruses 

The assignment of viral species through the last common ancestor allowed us to classify the known viral sequences by their putative host using the Virus–Host database [23], as described in [24]. Most of the sequences belong to viruses putatively infecting bacteria (60%) and invertebrates (~37%) (Figure 2C). Other relevant groups belong to known viruses that infect humans (~2.4%). Finally, there was a small contribution of viruses infecting plants, fungi, unicellular eukaryotes, non-mammal vertebrates and non-human mammals (Figure 2C). 

Most bacterial viruses belong to the *Picobirnaviridae* (55%), *Cystoviridae* (~4.4%) and *Levoviridae* (~0.2%) families, while invertebrate viruses are associated with the *Partitiviridae*-like sequences in the “unclassified RNA viruses ShiM-2016” group and *Totiviridae* family. Human viruses were composed exclusively of sequences from the *Reoviridae* family.

As discussed above, most of these viral families and their hosts have been reported in previous metagenomic wastewater studies [2,5,7,11,40]. Interestingly, the most abundant viruses in these studies belong to the *Virgaviridae* family and the Caudovirales order that infect plants and prokaryotes, respectively; however, these viruses appeared in low abundance in our sample [2,11]. The only RNA-based metagenomic study that was methodologically similar to our study also reported a high abundance of PBVs related sequences and rotaviruses [7]. The presence of PBVs sequences in the Trebal wastewater, which were genetically close to those found in animal feces, could be related to farm runoff, since the location of this WWTP (Figure 1) is outside the urban zone of Santiago and surrounded by many irrigation channels. Even though farm waste should not end up in the Trebal, whose exclusive purpose is the treatment of sewage from Santiago city, negligent handling of this waste could cause this result. 

The presence and high abundance of bacterial viruses (bacteriophages) is frequent in WWTPs. Their numbers are in part due to the release of phages that infect intestinal bacteria by human or animal defecation, but also from new infections of bacteria whose natural niche is the sewage ponds and sludge [42]. Bacterial RNA viruses are poorly understood in comparison with their DNA counterparts that are commonly found in sewage viral metagenomic studies [2,11]. The International Committee on Taxonomy of Viruses (ICTV) has only recognized two RNA bacteriophage families, the ssRNA family *Leviviridae* and the dsRNA family *Cystoviridae* [41]. Both families are represented by only 67 genomes in the NCBI RefSeq database (January 2020), which is low when compared to the 9661 viral DNA genomes in the same database. However, it has been proposed that the *Picobirnaviridae* is a new family of RNA bacteriophages based on the presence of bacterial ribosome binding sites (RBSs) upstream of the coding sequences, and also due to the lack of any consistent epidemiologic association with animal and human diseases [7,43,44]. 

In contrast to previous studies [2,41], most of the sequences associated with RNA bacteriophages that we found correspond to the ICTV-unrecognized *Picobirnaviridae* family and the *Cystoviridae* family, with only a small fraction associated with the *Leviviridae* family. All the known members of the *Cystoviridae* family infect *Pseudomonas* species, which are commonly present in eutrophic environments, such as sewage and the human body [45]. Therefore, the abundance of these viruses in the Trebal metagenome can expand the known sequence space associated with this family (only 10 genomes are currently available in the NCBI database) and contribute to a better understanding of the bacteriophage biology related to RNA genomes.

Invertebrate virus categories mostly include sequences of those that putatively infect annelids and arthropods, which was expected since these phyla have high densities in sewage stabilization ponds and aquatic environments of WWTPs [46,47]. In previous studies, a high prevalence of these invertebrate RNA viruses was not reported since only recently was their vast diversity discovered and their sequences made available in the databases [40].

Human viruses (which in Trebal correspond to *Rotavirus A* and *C*) are common in sewage and come from feces, urine, and respiratory secretions of infected hosts [9]. The most commonly identified viral pathogens in wastewater are *Adenovirus*, *Enterovirus*, *Hepatitis A* and *E* viruses, *Norovirus*, *Sapovirus*, and *Rotavirus A* [48]. These viruses are considered a potential public health risk because they are usually found at high concentrations in raw sewage, and their removal efficiency in WWTPs is not commonly assessed [8,49]. Moreover, since wastewater is composed of the excreta of thousands to millions of inhabitants, it is a representative sample that can be used for epidemiological surveillance purposes [5]. Therefore, metagenomic analysis of wastewater can highlight the presence of viral strains that circulate within a population, while enabling the discovery of new viruses that spread between humans and that are outside surveillance programs.

### 3.3. Tackling the Sewage RNA Viral Dark Matter through RNA-Dependent RNA Polymerase Analyses

**Figure 3:**
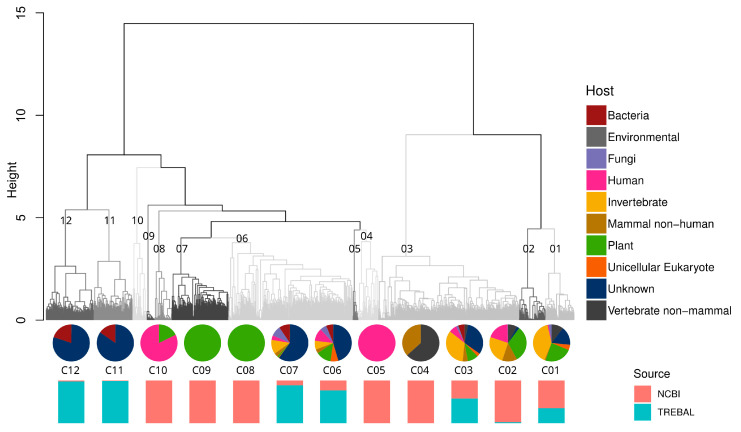
Hierarchical clustering analysis of 2266 RNA-dependent RNA polymerase (RdRP) predicted protein sequences from Trebal and NCBI RefSeq database based on Bray–Curtis amino acid distance (*k* = 2). Dendrogram was divided in 12 main cluster based on the “unrooted” dendrogram. Pie charts represent the frequency of sequences in each cluster classified by the putative host trough LCA algorithm. Bar charts represent the source (NCBI or Trebal) from which sequences were retrieved inside each cluster.

To uncover the untapped viral diversity present in the Trebal metagenome, which was not possible to recover through direct mapping of viral sequences against databases, we searched for RdRP homologous sequences using hidden Markov models (HMMs) for the protein. Subsequently, we estimated the genetic diversity of the RNA viruses using an alignment-free comparison of the retrieved RdRP sequences with those available in the NCBI RefSeq database (Figure 3). The RdRP is the most essential and conserved protein in RNA viruses [50]. It catalyzes RNA synthesis from RNA templates and is responsible for viral genome replication and transcription processes [49]. 

We identified 2180 predicted proteins as RdRPs using protein HMMs in the Pfam database [26]. Most of the RdRP sequences (65%) were classified as unknown since they do not align with any known protein in the NCBI nr database under standard cutoff parameters (*e*-value ≤ 1 × 10^−5^ and score ≥ 50). The latter is expected since the software that was used is designed to detect remote homologs in the most sensitive way, based on the strength of the underlying probability models (HMMs) [27]. The remaining 35% of the RdRPs corresponded to known viruses that putatively infect invertebrates (13%), bacteria (11%), unicellular eukaryotes (4%), fungi (3%), humans (2%), and plants (2%). 

Hierarchical clustering analysis based on the genetic distances of RdRPs showed 12 well-defined genetic clusters, five of which were formed exclusively by NCBI sequences and five of which were formed mostly by the Trebal sequences (Figure 3). Moreover, the sequences were clustered by a single host only in three of the 12 clusters (e.g., human viruses of cluster C05, and plant viruses of clusters C08-C09). Additionally, most of the RdRPs formed a continuous sequence space represented by highly heterogeneous clusters (e.g., C01-C03 and C06-C07). The latter feature was expected due to the orthologous nature of viral RdRPs and their degree of structural conservation inside the Riboviria realm [50,51]. Despite this, a closer inspection of the clusters with stricter cutoffs could reveal more specific associations.

The animal virus cluster C04 was formed exclusively by NCBI reference sequences inside the *Astroviridae* and *Coronaviridae* families, but no further precision regarding the host was feasible. Astroviruses are a commonly known cause of viral gastroenteritis in animals and humans [52]. Specifically, sequences of cluster C04 corresponded to avian *Astrovirus* associated with poultry and wild aquatic birds from a 2012 study in Asia [52]. Coronaviruses have a global distribution and infect a wide range of mammals and birds. They can cause respiratory and enteric infections that are usually mild, but severe infections of the respiratory system can develop, such as severe acute respiratory syndrome (SARS) and the infection currently responsible for a global pandemic, SARS-CoV-2 [53]. *Coronaviridae* sequences from cluster C04 were described in two studies from 2017 that investigated the viral population in bats in China and Vietnam [54,55]. Plant virus clusters C08-C09 exclusively represent NCBI reference sequences of the *Luteoviridae* (C08) and *Bromoviridae* (C09) families. Both viral families have a global distribution and are transmitted by specific aphid vectors [56,57]. These two viral families have a broad host range of genera within many plant families, causing necrosis in most of their hosts [56,57]. Finally, the human virus cluster C05 was formed exclusively by NCBI sequences of the *Norovirus* genogroup II (NoV GII). Noroviruses are a genetically diverse genus within the *Caliciviridae* family that can cause acute gastroenteritis in mammalian hosts [58]. Most of the human noroviruses belong to genogroups GI and GII, where NoV GII is usually the causal agent of acute gastroenteritis outbreaks [57,58]. These pandemic characteristics are probably related to the NoV GII epidemiology, which resembles that of *Influenza A* viruses, with the emergence of new variants every 2–3 years that replace the previously established variant [59]. 

Interestingly, bacterial viruses form two clusters, C11 and C12, which group *Picobirnaviridae* reference sequences from the NCBI, recovered from animal stools and Trebal new PBVs, together with unknown sequences that escaped our analyses using direct mapping against NCBI databases. This reflects the great diversity within the *Picobirnaviridae* family, which is not represented in current databases.

Taken together, our results show that metagenomic surveys of RNA viruses in sewage samples and the use of HMMs could uncover extraordinary viral diversity through the detection of remote homologs in these human-impacted environments. Additionally, the use of alignment-free genetic distances, such as the Bray–Curtis distance used here [28], can provide a reliable method for clusterization and classification based on related sequences for a large number of sequences, such as those generated by metagenomics methods.

### 3.4. High Phylogenetic Novelty of Picobirnaviruses in Trebal Sewage 

**Figure 4:**
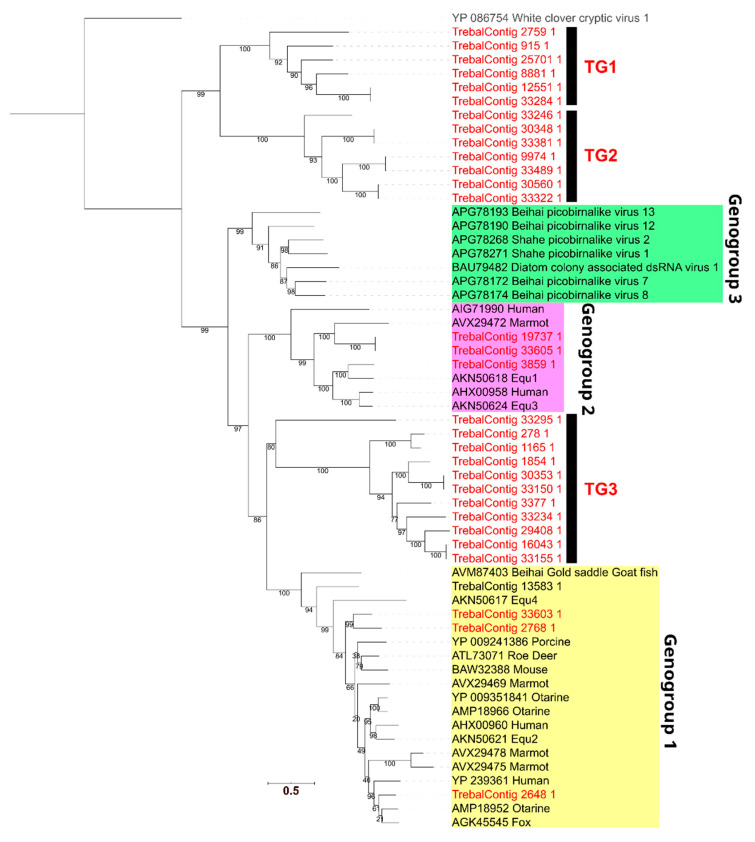
Maximum Likelihood phylogenetic reconstruction of 31 RNA-dependent RNA polymerase (RdRP) predicted proteins from *Picobirnaviridae* family. Node numbers indicate ultra-fast bootstrap values. RdRP sequence of White clover cryptic virus 1 was used as an outgroup and appeared in grey letters. The sequences characterized in the present study are reported in red letters. Scale bar: 0.5 aminoacid substitution per site.

To assess the novelty of the most abundant viral sequences observed in Trebal, namely those of the *Picobirnaviridae* family, we performed a phylogenetic analysis. First, we identified the RdRP protein sequences inside the *Picobirnaviridae* family and then filtered them by size to include only proteins ≥450 aa, which is the size of the smaller full-length PBV RdRP in the NCBI nr database. Next, we reconstructed a phylogenetic gene tree (Figure 4) using 31 RdRPs that met the filtering criteria and 25 reference PBV sequences. Our results show that seven Trebal sequences were associated with the known PBV genogroups [32] I and II associated with vertebrate stools. Interestingly, the rest of the environmental sequences (24) formed three separate monophyletic clades. Two of these exclusive Trebal monophyletic groups, TG1 and TG2 (13 sequences), could be considered sister clades of the known PBV genogroup three (GIII) associated with invertebrate samples [32]. In contrast, the third monophyletic group, TG3, which contains ten Trebal sequences, is between the PBV genogroups one (GI) and two (GII) [32], but closer to GII.

Therefore, TG3 can represent a highly divergent version of viruses that infect bacteria of the gastrointestinal tract from other vertebrates, such as domestic, farm or wild animals. This is highly probable since PBVs are ubiquitous in the feces of a vast range of animal species worldwide [39,60,61], including cattle, monkeys, dogs, cats, bats, horses, poultry and chickens [61].

For instance, PBV RdRPs sequences recovered from a metagenomic survey of bat stools in Cameroon showed a large group of highly divergent sequences that were closely related to the PBV GIII [62], as is the case of TG1 and TG2. This last evidence provides a probable origin for TG1 and TG2 since sewage ponds are known to be a feeding area for insectivorous bats [47].

New evidence of PBV sequences from sewage samples shows that sewage-recovered RdRPs were spuriously distributed between many PBV genogroups [7]. The latter reinforce the idea that PBVs do not infect mammals but are a new family of RNA bacteriophages, due to the consistent presence of bacterial RBSs upstream of the coding sequences [7,43,44]. To test this hypothesis, we searched for prokaryotic RBS motifs in the 31 RdRP sequences from the PBVs (Supplementary Table S3). We found that all except one of the full sequences (those not predicted in contig edges) contain an RBS, being AGGAGG and AGGAG, which are the most frequent motifs. This matches with other sewage PBVs that have the AGGAGG motif in 100% of the full RdRP [7]. This result is relevant because only prokaryotic viral families contain species whose genomes are highly enriched in RBS sequences [43,44]. Finally, PBVs have been assumed to be animal pathogens based on inferences from a few studies reporting the virus in diarrhea stool samples. However, they have not been cultured in animal cell lines, nor do they have any consistent epidemiologic association with diarrhea [43].

### 3.5. Dominance of Emergent Rotaviruses Genotypes in Trebal Sewage

*Rotavirus* species are classically defined by their major capsid protein VP6, whereas *Rotavirus* genotypes are defined based on their outer capsid proteins VP4 and VP7 [63]. In Chile, over the last ten years, globally common *Rotavirus* genotypes, such as G1P(8), G4P(8), G2P(4) and G9P(8) have alternated in their dominance, while emerging genotypes, such as G8P(8), have only recently been reported [63].

**Figure 5:**
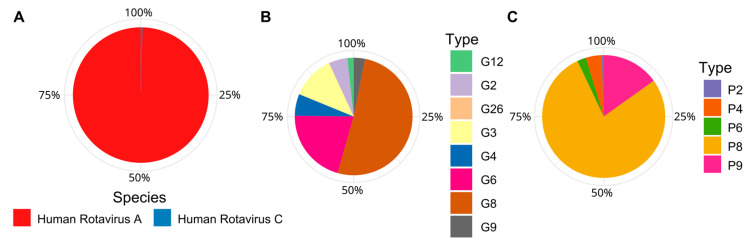
Relative abundances of Human *Rotavirus* species and genotypes inferred from Pfam annotated VP4, VP6, and VP7 genes and classified by local alignment (BLASTn) to NCBI nt database. Sequences were normalized by gene length. (**A**) Relative abundance of Human *Rotavirus* species based on VP6. (**B**) Relative abundance of Human *Rotavirus* G types based on VP7. (**C**) Relative abundance of Human *Rotavirus* P types based on VP4.

Here, using Pfam annotation of the Trebal predicted proteins, we recovered 30 VP4, 29 VP6 and 27 VP7 human *Rotavirus* proteins. Local alignment-based classification (Figure 5) shows that the most abundant *Rotavirus* species belongs to human *Rotavirus A*, which is the most common cause of hospitalization due to viral gastroenteritis worldwide [63]. However, it is interesting to note that the presence (in low abundance; 0.4%) of human *Rotavirus C* sequences that are closely associated to Asian strains, have, to date, only been reported in Chile through personal communication. Likewise, the most abundant *Rotavirus* genotypes were G8 (51%), G6 (21%) and G3 (12%) and P8 (78%) and P9 (15%). The segmented nature of *Rotavirus* genomes does not allow us to infer the G-P genotype classification because we do not know which combinations of VP4 and VP7 were inside the same viral particle. However, it is highly probable that the most abundant G genotypes were combined with the most abundant P genotypes—for example, G8P(8), G6P(8) or G6P(9). 

Of these genotypes, G8P(8) was recently reported as an emergent *Rotavirus* strain in a medium-sized city near Santiago between 2016 and 2018 [63]. This strain has not been previously reported in South or North America, but has a highly similar identity to sequences detected between 2010 and 2016 in Asia [63]. In the same line of evidence, the G8 sequences found in Trebal also shared a 99% nucleotide identity with sequences detected in 2013 in Thailand [64] and between 2017 and 2018 in Japan [65].

The *Rotavirus* G6 genotype is also an emergent strain, with most of the reports concentrated in Europe between 2010–2013, but spurious circulation has been reported since the 2000s [66,67]. In our analysis, the G6 recovered from the Trebal sequences are closely related (99% nucleotide identity) to a G6P(9) strain detected in Germany in 2014 and to a G6P(6) strain detected in Belgium in 2002; yet, to our knowledge, this is the first report of *Rotavirus* G6 in Chile. 

These results emphasize the relevance of sewage viromes as epidemiological surveillance tools. Likewise, our study demonstrates the advantage of using viral metagenomics for this task, which, despite using short sequences, can deliver reliable results that can later be confirmed by other methodologies. The latter point is especially relevant for *Rotavirus* since surveillance based on PCR has shown serious bias related to primer specificity [63]. These results are especially significant for Chile, as it is one of the few South American countries that has not implemented a national *Rotavirus* vaccination program [63]. Therefore, identification of genotypes that have not been previously reported in Chile represents a first step in *Rotavirus* prevention. Furthermore, this can be used to generate valuable information to improve or implement new vaccination programs against this disease worldwide.

This study explored the use of viral metagenomics to discover RNA viruses in sewage and it is the first insight into the wastewater virosphere from a large city in South America. We have demonstrated the utility of metaviromics for the discovery of new groups and viral genotypes associated with known families. This is especially important due to the underrepresentation of PBVs in databases and the presence of uncommon *Rotavirus* genotypes that are usually beyond the range of PCR-based surveillance. Consequently, viral metagenomics can be used for the exploration and surveillance of emergent viruses, to better design new viral markers for diagnoses and routine epidemiological work, and for the implementation of national vaccination programs.

## Supplementary Materials

The following are available online at https://www.mdpi.com/1999-4915/12/9/1050/s1, Supplementary Table S1: Summary information about sequencing depth, quality filtering, read mapping and assembly of Trebal RNA viral metagenome, Supplementary Table S2: Relative abundances of viral families classified by LCA algorithm trough local alignment to NCBI nr database, Supplementary Table S3: Frequency of ribosomal binding site (RBS) motifs found in 31 Picobirnaviridae RNA-dependent RNA polymerase (RdRP) predicted proteins.
