# Supplementary material for: Metagenomic Insights into the Sewage RNA Virosphere of a Large City
Source: Viruses. 2020 Sep 21;12(9):1050. doi: 10.3390/v12091050 (PMC7551614; doi:10.3390/v12091050)
Supplement: Supplementary file 1 [file viruses-12-01050-s001.pdf]

Supplementary Material

Metagenomic insights into the sewage RNA virosphere of a large city.

Sergio Guajardo-Leiva[1], Jonás Chnaiderman[2], Aldo Gaggero[2,*] and Beatriz Díez[1,3,*].

[1]Department of Molecular Genetics and Microbiology, Pontificia Universidad Católica de Chile, Santiago, Chile.

[2]Programa de Virología, ICBM, Facultad de Medicina, Universidad de Chile, Chile.

[3]Center for Climate and Resilience Research (CR)2, Chile.

*** Correspondence:**

Beatriz Díez

bdiez@bio.puc.cl

Aldo Gaggero

agaggero@uchile.cl

Supplementary Table S1: Summary information about sequencing depth, quality filtering, read mapping and assembly of Trebal RNA viral metagenome.

Feature	Numbers
Total reads	46,303,799
Quality filtered reads	44,979,942
Assembled contigs	62,164
Reads aligned to contigs	34,803,702
Predicted proteins in contigs	72,313
Proteins aligned to NCBI nr	8,350
Reads in NCBI nr aligned proteins	5,069,442

Supplementary Table S2: Relative abundances of viral families classified by LCA algorithm trough local alignment to NCBI nr database.

Family	Relative Abundance
Picobirnaviridae	55.035
Partitiviridae-like (ShiM-2016)	25.115
Totiviridae	6.788
Cystoviridae	4.402
unclassified viruses	2.623
Reoviridae	2.369
Totiviridae-like (ShiM-2016)	1.612
Tombusviridae-like (ShiM-2016)	0.905
Virgaviridae	0.293
Leviviridae	0.220
Partitiviridae	0.135
Endornaviridae	0.114
Chrysoviridae	0.110
unclassified dsRNA viruses	0.079
Myoviridae	0.053
Birnaviridae	0.043
environmental samples viruses	0.027
unclassified bacterial viruses	0.015
Betaflexiviridae	0.011
Picornavirales	0.008
unclassified DNA viruses	0.008
ssRNA viruses	0.007
Alphaflexiviridae	0.006
Siphoviridae	0.005
Narnaviridae	0.004
Bromoviridae	0.003
Microviridae	0.003
Tombusviridae	0.002
Podoviridae	0.001
unclassified Caudovirales	0.001
Mimiviridae	0.001
Megabirnaviridae	0.001
Hypoviridae	0.001

Suppelentary Table S3: Frequency of Ribosomal binding site (RBS) motifs found in 31 Picobirnaviridae RNA dependent RNA polimerase (RdRP) predicted proteins.

RBS-Motif	Frequency
AGGAGG	6
AGGAG	5
4Base/6BMM	2
AGGA	1
GGA/GAG/AGG	1
GGAG/GAGG	1
GGAGG	1
GGxGG	1
None	1
None in edges	12
